# *Lactobacillus* Probiotic Strains Differ in Their Ability to Adhere to Human Lung Epithelial Cells and to Prevent Adhesion of Clinical Isolates of *Pseudomonas aeruginosa* from Cystic Fibrosis Lung

**DOI:** 10.3390/microorganisms11071707

**Published:** 2023-06-29

**Authors:** Giovanna Batoni, Esingül Kaya, Elisa Catelli, Sabrina Quinti, Matteo Botti, Alessandro De Carli, Marta Bianchi, Giuseppantonio Maisetta, Semih Esin

**Affiliations:** 1 Department of Translational Research and New Technologies in Medicine and Surgery, University of Pisa, 56123 Pisa, Italy; e.kaya@studenti.unipi.it (E.K.);; 2 Cystic Fibrosis Supporting Service, Azienda USL Toscana Nord-Ovest, 57128 Livorno, Italy; 3 Department of Medical Biotechnologies, University of Siena, 53100 Siena, Italy

**Keywords:** *Lactobacillus*, cystic fibrosis, *Pseudomonas aeruginosa*, adhesion assay, immunomodulation, probiotics, exclusion assay

## Abstract

The field of probiotic applications is rapidly expanding, including their use for the control of respiratory tract infections. Nevertheless, probiotics ability to colonize the lung environment and to compete with pulmonary pathogens is still a poorly investigated research area. In this study, we aimed to evaluate the adhesion ability of a number of commercial probiotic strains to the human lung epithelial cell line A549. Furthermore, we assessed probiotic ability to prevent host cell adhesion of one of the major lung pathogens in cystic fibrosis, *Pseudomonas aeruginosa*, and to reduce the pathogen-induced inflammatory response of human peripheral blood mononuclear cells (PBMCs) in terms of cytokine release. *Lactobacillus acidophilus* displayed the highest adhesion ability to A549 cells evaluated as percent of adhered bacteria compared to the inoculum. In agreement with such an observation, *L. acidophilus* was the most efficient in preventing adhesion to A549 cells of a *P. aeruginosa* isolate from CF sputum. Three-color fluorescence labeling of A549 cells, *P. aeruginosa*, and *L. acidophilus*, and confocal microcopy image analyses revealed a likely exclusion effect played by both live and UV-killed *L. acidophilus* towards *P. aeruginosa*. Such results were confirmed by CFU count. When co-cultured with PBMCs, both live and UV-killed *L. acidophilus* reduced the amount of IL-1β and IL-6 in culture supernatants in a statistically significant manner. Overall, the results obtained point to *L. acidophilus* as an interesting candidate for further studies for a potential aerogenous administration to control *P. aeruginosa* infections.

## 1. Introduction

Nowadays, the use of probiotics, i.e., live microorganisms that when administered in adequate amounts confer a health benefit to the host, is a measure increasingly taken into consideration to prevent/cure a number of human diseases [1,2]. In particular, the possibility to exploit such microorganisms to compete with and control highly pathogenic bacterial strains is an attractive approach in the era of multi-drug resistance, when the therapeutic potential of conventional antibiotics is rapidly vanishing. These health-boosting living microorganisms mostly belong to the Gram-positive bacteria, with the *Lactobacillus* genus being one of the most widely used [3].

Recent evidence highlights that administration of *Lactobacillus* probiotic strains can exert protective effects not only in their traditional field of application, i.e., the gut, but also in distant body areas such as the respiratory tract, reducing the rate of pulmonary infections, and/or ameliorating respiratory symptoms [3]. For instance, it has been reported that the oral administration of *L. casei* CRL 431 to young mice enhances the phagocytic activity of alveolar macrophages and the lung clearance of *Pseudomonas aeruginosa* [4]. The same strain was also reported to increase the resistance to *Streptococcus pneumoniae* respiratory infection in malnourished mice [5]. A randomized, double-blind, placebo-controlled pilot study in intensive care unit patients demonstrated that the occurrence of *P. aeruginosa* respiratory colonization and/or infection was significantly delayed in the patients administered with lactobacilli when compared to the control group [6], while a cocktail of probiotics, including lactobacilli, was shown to reduce the incidence of ventilator-associated pneumonia in children, as well as the colonization rate of potentially pathogenic bacteria such as *Klebsiella* and *Pseudomonas* [7]. There are also examples of the protective role of probiotics against serious viral respiratory infections, including COVID-19 [8,9], although reports not supporting a role of probiotics in preventing respiratory infections exist as well [10,11]. The question of the clinical efficacy of probiotic supplementation to prevent respiratory infections is therefore still open, demanding in-depth studies at the level of preclinical and clinical research to clarify the possible mechanisms involved in probiotic action. 

Over the last decade it has become progressively clear that the gut microbiota play a major role in mediating respiratory outcomes through mechanisms attributable to the “gut-lung axis” [12]. These mechanisms include migration of microbiota-activated immune cells and cytokines from the intestine to the lung through the systemic circulation; passage of microbial-derived metabolites with immune-modulating activity (e.g., short chain fatty acids-SCFA) from the gut to the lung tissue; and direct transfer of intestinal bacteria to the lung through circulation or via gastroesophageal reflux [3]. This latter mechanism is supported by studies demonstrating that lung colonization/infection by microbiome components or pathogenic strains is often preceded by gut colonization, pointing to the intestine as a reservoir of microorganisms that can spread to other organs [13,14].

Respiratory infections are a hallmark of cystic fibrosis (CF), a heritable, autosomal disease caused by mutations in the cystic fibrosis transmembrane conductance regulator (CFTR) gene [15]. Due to mutations in the CFTR gene, patients experience an altered secretion of chloride and bicarbonate that causes, among other symptoms, the production of an abnormally thick mucus in the lungs that, in turn, hampers muco-ciliary clearance and favors bacterial persistence [16]. In this context, *P. aeruginosa* represents one of the major pathogens leading to lung injury and decline in respiratory function in CF patients [17]. Probiotic administration to prevent/treat pulmonary infections is a measure under evaluation in CF, with both oral and respiratory administration being investigated [18,19]. Commercial strains of probiotics are mostly administered via the oral route and, therefore, are selected for their ability to pass the gastric barrier, colonize the intestinal mucosa, and eventually compete with pathogens of the gastro-intestinal tract. These same properties do not necessarily coincide with those required to colonize and survive in the lung or exert a protective role against respiratory pathogens such as *P. aeruginosa*. Thus, studies to identify the best probiotics to compete with CF pathogens or to colonize the CF lung ecological niche are needed to lay rational foundations for probiotic supplementation in CF. Interestingly, recent evidence suggests that the beneficial effects of probiotics go beyond their viability and that dead bacteria (also referred to as paraprobiotics) or probiotic-derived components (also referred to as postbiotics) may represent valid alternatives to live probiotics in terms of safety and ease of production and storage [20]. Nevertheless, several aspects related to the bioactivities of postbiotics and paraprobiotics remain unexplored or poorly understood. In this framework, the aim of this study was to screen a number of commercial *Lactobacillus* strains in the context of pulmonary environment by: (i) testing in vitro their adhesion properties to the human lung epithelial cell line A549; (ii) evaluating the ability of *Lactobacillus* strains to prevent adhesion to A549 cells of *P. aeruginosa* lung isolates from CF patients by developing a three-color confocal microscopy exclusion assay; (iii) investigating the immunomodulatory properties of the *Lactobacillus* strains by assessing the release of pro-inflammatory cytokines by human peripheral blood mononuclear cells (PBMCs) stimulated with lung isolates of *P. aeruginosa* in the presence or absence of lactobacilli; and (iv) comparing the bioactivities of live and dead probiotics at the host–pathogen interface. 

Overall, the results obtained demonstrated a diversity in the adhesion properties of various *Lactobacillus* strains to lung epithelial cells. *L. acidophilus* emerged as the strain with the higher adhesive properties. *L. acidophilus* itself also showed the ability to inhibit the adherence of *P. aeruginosa* to lung epithelial cells (by a likely exclusion effect) and to reduce the release of pro-inflammatory cytokines from human PBMCs stimulated with *P. aeruginosa*. Interestingly, no major difference was observed between live and dead *L. acidophilus* in carrying out these effects, opening suitable possibilities for probiotic therapeutical intervention in vulnerable subjects. 

## 2. Materials and Methods

### 2.1. Bacterial Strains and Growth Conditions

The probiotic strains tested in this study were isolated from products commercially available in Italy (e.g., dietary supplements) and identified by MALDI-TOF MS (Bruker Daltonics, Macerata, Italy). Strains, the manufacturer (in parenthesis), and the corresponding codes used throughout the study were as follows: *Lacticaseibacillus rhamnosus* (Microbiosys, Sanofi Aventis, Milan, Italy), LRm; *L. rhamnosus* (Dicoflor, AG Pharma, Roma, Italy), LRd; *L. rhamnosus* ATCC 7469, LRa; *L. paracasei* (Biotics G, Burgerstein, Rapperswil-Jona, Switzerland), LPC; *Limosilactobacillus fermentum* (Urotab, Unifarco Spa, Belluno, Italy), LF; *Lactiplantibacillus plantarum* (Biotics G), LP; *Lactobacillus acidophilus* (Nature’s Bounty, Green Remedies Spa, Padova, Italy), LA; *L. gasseri* (Bayer Spa, Milan, Italy), LG. For preparation of stock cultures, isolated colonies of lactobacilli grown on the De Man Rogosa and Sharpe agar (MRSA, Thermo Fisher Diagnostics Spa, Rodano, Italy) were picked up and inoculated in De Man Rogosa and Sharpe broth (MRSB, Thermo Fisher, Monza, Italy). After overnight incubation at 37 °C in shaking conditions, cultures were divided in aliquots and stored at −80 °C until use. 

Two *P. aeruginosa* strains, named CF1 and CF4, and exhibiting a non-mucoid and a mucoid phenotype, respectively, were used in the study. They are part of a collection of clinical isolates stored at the Microbiology Section of the Department of Translational Research and new Technologies in Medicine and Surgery of the University of Pisa. They were isolated from the sputum of CF patients during the course of routine follow-ups, and identified by MALDI-TOF MS.

### 2.2. Adhesion Assay of Lactobacilli to the A549 Human Lung Adenocarcinoma Cell Line 

A549 cells (LGC Standards, Milan, Italy) were seeded in 96-well plates at a cell density of 25,000/well in DMEM High glucose medium added with 10% fetal bovine serum (FBS) and 2 mM L-glutamine (Euroclone) (complete DMEM). After 24 h, A549 cells in confluence were incubated with a panel of eight commercial lactobacilli at a multiplicity of infection (MOI) of 10:1 (bacteria per cell). After 2 h incubation at 37 °C to allow adhesion, the monolayers were washed to remove non-adherent bacteria, cells lysed with 0.1% Triton X-100 in water (Merck, Milan, Italy), and cell lysates plated in serial dilutions on MRSA for colony forming unit (CFU) counting.

Based on the results obtained, *L. acidophilus* and *L. plantarum* were selected for a more in-depth investigation. To this end, the two strains were incubated with A549 cells at different MOIs (from 1:1 to 1000:1) to identify optimal adherence conditions. Following three washes to remove non adherent bacteria, CFU counts were performed as above. In parallel, cell viability was assessed using the trypan blue exclusion viability assay (see Section 2.3). 

### 2.3. Trypan Blue Dye Exclusion Viability Assay

The viability of A549 cells was assessed with the trypan blue dye exclusion test following a 2 h adhesion assay with *L. acidophilus* and *L. plantarum,* or incubated alone as previously described [21]. To this end, following the adherence assay, the A549 monolayers were washed three times and detached by 3 min. treatment with Trypsin/EDTA solution (Euroclone SpA, Pero, Milan, Italy). Following a wash with phosphate-buffered saline (PBS, Euroclone), the cells were resuspended and diluted 5 times with 0.4% trypan blue (Euroclone). An aliquot of the suspension was inserted into a Burker counting chamber (Merck, Milan, Italy) and observed under 400× magnification with a light microscope (Olympus CH20BIMF200, Olympus Italy, Segrate, Milan, Italy). Two operators independently counted live (clear) and dead (blue) cell numbers from six different fields. The mean values ± SEM were reported.

### 2.4. P. aeruginosa Exclusion Assay via Confocal Microscopy

In a first set of experiments, a one-color exclusion assay was performed by labeling *P. aeruginosa* CF1 strain with the green fluorescent lipophilic dye PHK67 (Merck) that binds bacterial cell membranes. To this end, 20 × 10^6^ bacteria/mL were incubated with 5 × 10^−6^ M lipophilic dye for 8 min. at room temperature in shaking conditions. The staining was stopped by adding 1:1 FBS for 1 min. Bacteria were then washed twice and resuspended in complete DMEM. In parallel, 5 selected lactobacilli strains were added to confluent monolayers of A549 cells at a MOI of 100:1 bacteria per cell. Following 2 h of incubation at 37 °C in humidified atmosphere with 5.5% CO_2_, non-adhered lactobacilli were removed by three gentle washes with warm PBS. *P. aeruginosa* labeled as above was added to the monolayers at a MOI of 10:1. A549 monolayers incubated with *P. aeruginosa* in the absence of lactobacilli were also established as positive controls. After an additional hour of incubation and subsequent washes with PBS, the fluorescent images of the monolayers were acquired by confocal microscopy using the Operetta CLS High-Content Analysis System (PerkinElmer Inc., Waltham, MA, USA) of the Center for Instrument Sharing of the University of Pisa. Images were then analyzed using Harmony software (version 4.9, PerkinElmer Inc., USA) and total fluorescence intensity of the observed fields was calculated as number of fluorescent objects × mean fluorescence intensity.

In a second set of experiments, a three-color exclusion assay was developed by using three long-tracking fluorescent dyes to differentially label *L. acidophilus*, *P. aeruginosa*, and A549 epithelial cells. To this end, adherent A549 cells in 100% confluence were labeled in blue with the Biotracker^TM^ 400 according to manufacturer’s instructions (Thermo Fisher). After that, the exclusion assay was performed as described above following the staining of *L. acidophilus* and *P. aeruginosa* with the orange fluorescence dye PKH26 (Merck) and with the green fluorescence dye PHK67, respectively. In some experiments, *L. acidophilus* killed by exposure to UV-light for 1 h was used instead of live *L. acidophilus*. The efficacy of the killing procedure, assessed by plating of the UV-light exposed bacteria onto MRSA, was 100% in all experiments.

### 2.5. P. aeruginosa Exclusion Assay via CFU Count

The exclusion effect of *L. acidophilus* on *P. aeruginosa* adhesion to A549 monolayers was also evaluated using CFU counts. To this end, un-labeled lactobacilli (*L. acidophilus* and *L. rhamnosus*) and *P. aeruginosa* (CF1 and CF4) were used in exclusion assays as described above. Following the incubation with *P. aeruginosa*, cells were washed to remove un-bound bacteria and lysed by adding 0.1% Triton X-100 solution for 10 min. Following a wash with PBS at 4000× *g* for 5 min, bacteria were resuspended in PBS, serially diluted, and plated onto cetrimide agar (Merck) to determine CFU counts.

### 2.6. Peripheral Blood Mononuclear Cell (PBMC) Isolation

Blood was drawn from donors attending the Transfusion center of Pisa University Hospital or from healthy volunteers after an informed consent was obtained. The study was conducted in accordance with the Declaration of Helsinki, and the protocol was approved by the local Ethical Committee (Comitato Etico Area Vasta Nord-Ovest, CEAVNO, Protocol 34743, 28 June 2018). PBMCs were isolated from buffy coats by standard gradient separation as described previously [21]. Briefly, buffy coats were diluted 1:1 with PBS, 10% sodium citrate (*v*/*v*) (Merck). Cell suspensions were layered on a density gradient (Lymphoprep, Cedarlane, ON, Canada), and subjected to 20 min centrifugation at 160× *g* at room temperature. Afterwards, platelets in the supernatant were gently removed without disturbing the mononuclear layer at the interface. After a further centrifugation at 800× *g* for 20 min, PBMCs were collected from the interface and washed three times in PRMI. Finally, PBMCs were resuspended in complete RPMI, replacing the fetal bovine serum with 10% heat-inactivated autologous plasma.

### 2.7. Co-Culture of PBMCs with P. aeruginosa and Lactobacilli

PBMCs (1 × 10^6^ PBMC/mL, 1 × 10^5^ PBMC/well) resuspended in RPMI 1640 supplemented with 10% FBS and 2 mM L-glutamine (Euroclone) (complete RPMI) were added to each well of a 96-well plate. Bacteria were diluted in complete RPMI to obtain a MOI of 10:1 for lactobacilli and 1:1 for *P. aeruginosa*, respectively, and added to the PBMCs. PBMCs incubated without bacteria or incubated with *P. aeruginosa* only represented negative and positive controls, respectively. In further experiments, live and UV-killed *L. acidophilus* were used in parallel. PBMC:bacteria co-cultures were incubated at 37 °C in 5.5% CO_2_ for 4 h. Following incubation, PBMC viability was evaluated with a trypan blue dye exclusion assay (see Section 2.3 above). Supernatants from each experimental condition were sterile filtered (0.22 µm), aliquoted, and stored at −20 °C until cytokine determination.

### 2.8. Quantification of Cytokines Released in Culture Supernatants

The amount of pro-inflammatory cytokines IL-1β and IL-6 were measured in the supernatants with a flow-cytometer-based multibead capture assay (LEGENDplex^TM^ Multi-Analyte Flow Assay Kit, BioLegend Inc., San Diego, CA, USA) following manufacturer’s instructions. Sensitivities of the assay were as follows: IL-1β, 0.65 ± 0.47 pg/mL; IL-6, 0.97 ± 1.46 pg/mL. Acquisition of the samples was performed with a BD Accuri C6 flow cytometer (BD Biosciences, Milan, Italy). Data were analyzed with the LegendPlex v8.0 Software (BioLegend Inc.), and the amount of cytokines was calculated based on a standard curve. Results were expressed as ng/mL.

### 2.9. Statistical Analysis

The statistical significance of the data was assessed using GraphPad In Stat (version 3.06, GraphPad Software Inc., La Jolla, CA, USA) using one-way analysis of variance (ANOVA) followed by Tukey–Kramer post hoc test, Student’s *t*-test, and non-parametric Wilcoxon signed-ranks test. A level of *p* < 0.05 was considered statistically significant. 

## 3. Results

### 3.1. Adhesion Ability of Lactobacilli to the Human Lung Epithelial Cell-Line A549

**Figure 1:**
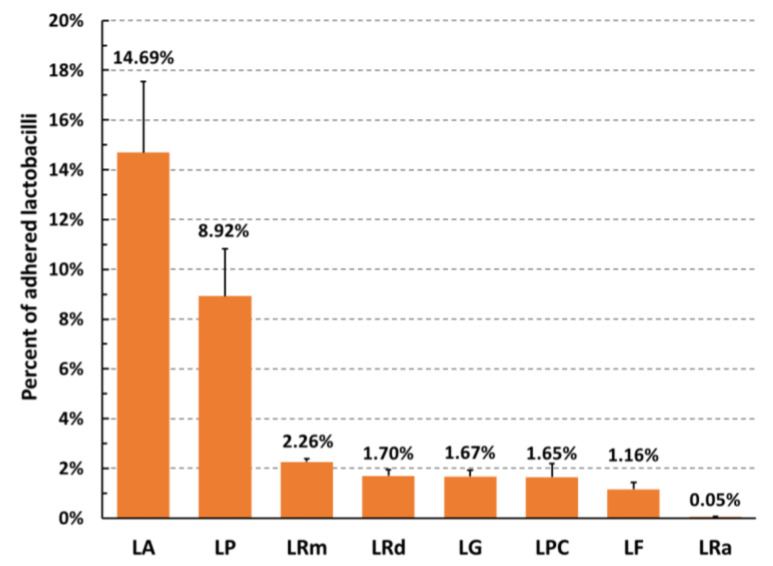
Adhesion ability of different strains of lactobacilli to the lung epithelial cell line A549 (MOI 10:1 bacteria:cell). Percent of adhered bacteria as compared to the inoculum. Mean values ± SEM of four independent experiments are depicted. LA: *L. acidophilus*; LP: *L. plantarum*; LRm: *L. rhamnosus* (Microbiosys); LRd: *L. rhamnosus* (Dicoflor); LG: *L. gasseri*; LPC: *L. paracasei*; LF: *L. fermentum*; LRa: *L. rhamnosus* (ATCC).

Adhesion ability to host cells is a classical selection criterion for potential probiotic bacteria. Therefore, we aimed to evaluate adhesion ability of a panel of eight commercial strains of lactobacilli in the context of the pulmonary environment, taking as a model the human lung epithelial cell line A549. As shown in Figure 1, the various strains of lactobacilli showed a variable ability to adhere to human lung epithelial cells, evaluated as the percentage of bacteria recovered after adherence with respect to the inoculum. LA showed the highest adhesive capacity with a percentage of about 15%, followed by LP (8.9%) and LRm (2.3%). The adhesion abilities of LRm, LG, LPC, LF, and LRa, were all below 2%.

### 3.2. Lactobacilli Effect on Host-Cell Viability

**Figure 2:**
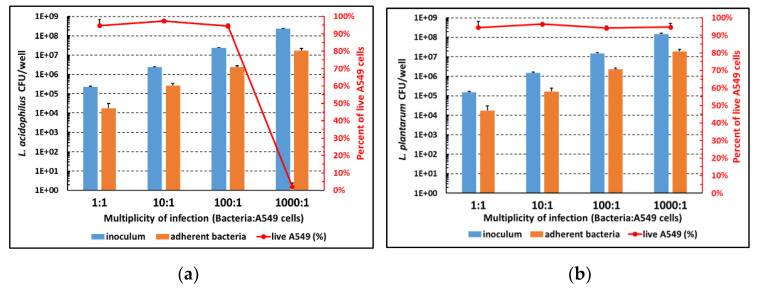
Adhesion capacity to A549 cells and cytotoxic potential of LA (**a**) and LP (**b**) at different bacteria:cell ratios. Left axis: number of adhered bacteria; right axis: A549 cell vitality. Mean values ± SEM of four independent experiments in duplicates are depicted.

Based on adherence results, LA and LP were selected for a more in-depth investigation; to this end, the two strains were incubated with A549 cells at different MOIs (from 1:1 to 1000:1) to identify optimal adherence conditions. Following three washes to remove non-adherent bacteria, CFU counts were performed. In parallel, cell viability was assessed using the Trypan blue exclusion viability assay. As seen in Figure 2, the number of adherent bacteria of both strains progressively increased as the inoculum increased. Cell viability (red line) remained high (greater than 90%) under all experimental conditions except for the 1000:1 MOI of LA. Therefore, a MOI of 100:1 was chosen for the subsequent experiments as the best compromise between adherence and cell viability.

### 3.3. Ability of Different Strains of Lactobacilli to Prevent P. aeruginosa Adhesion to A549 Cells via Confocal Microscopy

**Figure 3:**
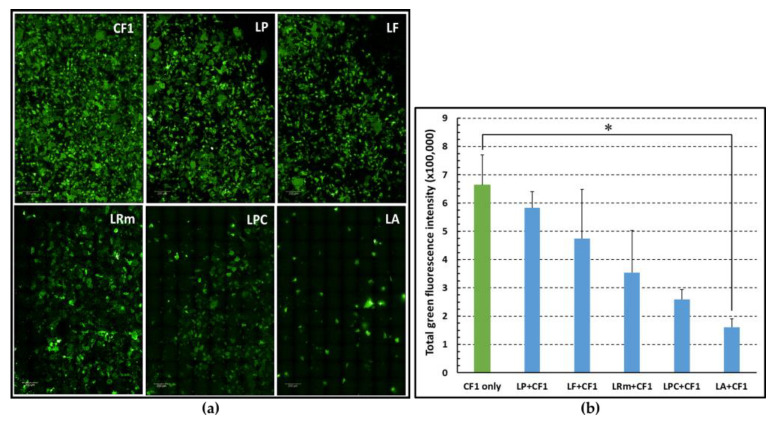
Inhibition of *P. aeruginosa* adherence (strain CF1) to A549 cells pre-incubated or not with five strains of lactobacilli. (**a**) 63× confocal images of green fluorescent CF1 adhered to A549 cells pre-incubated for 2 h with the indicated strains of lactobacilli or in the absence of lactobacilli (CF1); images from a representative experiment are shown. (**b**) Total green fluorescence intensity analyses of CF1 adherence to A549 cells pre-incubated with different strains of lactobacilli or incubated for 1 h with CF1 only. * *p* < 0.05, One-way analysis of variance test followed by Tukey–Kramer multiple comparisons test. Mean values ± SEM of four independent experiments in duplicates are depicted.

At the MOI of 100:1, exclusion experiments were carried out to evaluate the ability of the different lactobacilli to inhibit the adherence of a clinical isolate of *P. aeruginosa* (strain CF1) to the A549 cells. As shown in Figure 3, compared to the control (i.e., the CF1 clinical isolate incubated in the absence of lactobacilli, green bar), all the strains of lactobacilli analyzed caused a reduction in the adhesion of *P. aeruginosa*, evaluated as total fluorescence intensity, although at different extents. In agreement with the adhesion data, LA was the one causing the greatest and statistically significant reduction in fluorescence, followed by LPC and LRm.

### 3.4. Three-Label Host-Cell Adhesion Assay to Assess the Exclusion Effect Exerted by LA on P. aeruginosa Adhesion 

**Figure 4:**
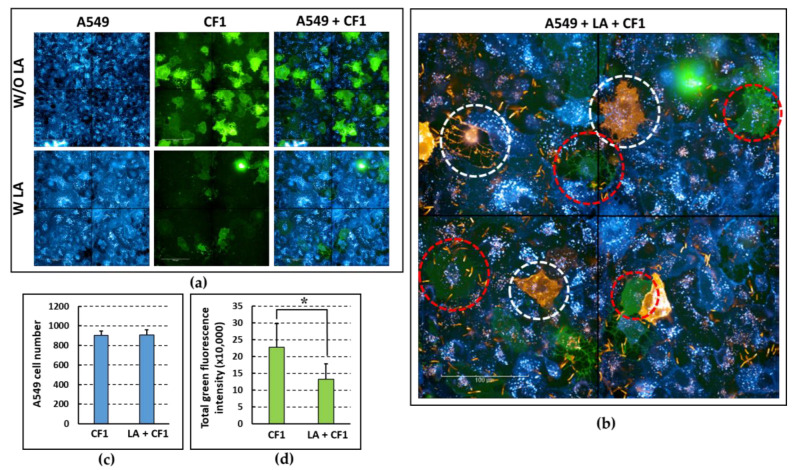
LA ability to inhibit *P. aeruginosa* (CF1) adhesion to A549 cells demonstrated via triple-fluorescence staining and confocal microscopy. (**a**) Biotracker^TM^400 blue staining of A549 cells (A549), PHK67 green staining of *P. aeruginosa* (CF1), and Biotracker^TM^400/PHK67 merged staining (A549 + CF1), in the presence (W LA) or absence (W/O LA) of LA. (**b**) Merged staining of A549 (Biotracker^TM^400, blue), CF1 (PHK67, green), and LA (PKH26, orange); white circles: A549 cells with a high number of adherent lactobacilli; red circles: A549 cells with few adherent lactobacilli. (**c**) Quantitative evaluation of number of A549 cells (blue fluorescence) exposed to *P. aeruginosa* only (CF1) or to LA followed by exposure to CF1 (LA + CF1). (**d**) Quantitative evaluation of green fluorescence intensity of CF1 incubated with A549 cells only (CF1) or with A549 cells pre-incubated with LA. * *p* < 0.05 Student’s *t* test.

The interaction between LA, *P. aeruginosa* and epithelial cells was further investigated through the development of a 3-label host-cell adhesion assay. To this end, three long-tracking fluorescent dyes were used to differentially mark A549 cells in blue, lactobacilli in orange, and *P. aeruginosa* in green. The exclusion assay was then performed as described above. Samples were observed with confocal microscopy and the fluorescence intensity was assessed using dedicated software. Figure 4 shows the data from a representative experiment. The levels of blue fluorescence, i.e., the number of host cells, were comparable in the absence and in the presence of LA (Figure 4a,c). This confirmed that lactobacilli, at the MOI used, do not exert a cytotoxic effect or a negative impact on the adhesion of the monolayers. In the presence of LA, the green fluorescence of *P. aeruginosa* was significantly reduced (Figure 4a,d), suggesting an exclusion effect played by this strain towards the CF1 clinical isolate. The exclusion effect played by lactobacilli is best appreciated in the 3-colour image (Figure 4b) where the white circles indicate A549 cells with a high number of adherent lactobacilli, while the red circles indicate cells with few adherent lactobacilli, which were also those on which *P. aeruginosa* adhered the most.

### 3.5. Ability of LA and LRm to Prevent P. aeruginosa Adhesion via CFU Count

**Figure 5:**
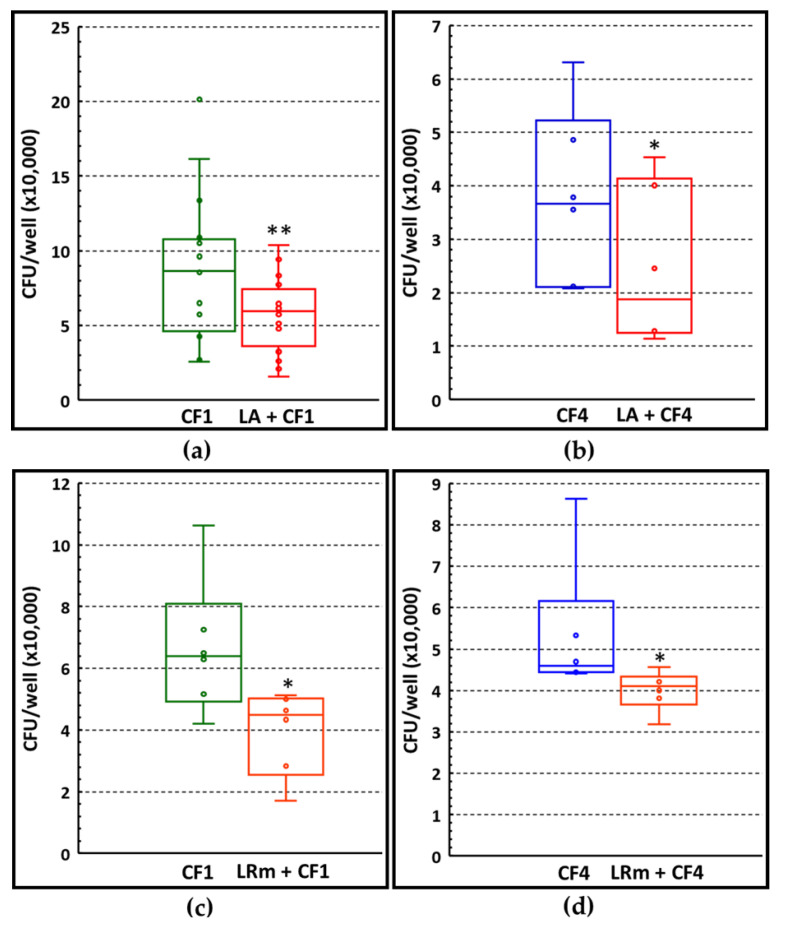
Ability of lactobacilli to prevent *P. aeruginosa* adhesion to A549 cells via CFU count. (**a**) CFU count of *P. aeruginosa* CF1 strain (non-mucoid) following incubation with A549 cells pre-incubated or not with LA. (**b**) CFU count of *P. aeruginosa* CF4 strain (mucoid) following incubation with A549 cells pre-incubated or not with LA. (**c**) CFU count of *P. aeruginosa* CF1 strain following incubation with A549 cells pre-incubated or not with LRm. (**d**) CFU count of *P. aeruginosa* CF4 strain (mucoid) following incubation with A549 cells pre-incubated or not with LRm. * *p* < 0.05; ** *p* < 0.01, Wilcoxon matched-pairs signed-ranks test, n = 6 to n = 15.

The exclusion effect played by lactobacilli versus *P. aeruginosa* was further investigated in terms of CFU count. In addition to LA, LRm was also tested, as this latter strain has demonstrated a good ability to grow in conditions mimicking the CF lung environment, and to exert an antibiofilm effect against *P. aeruginosa* isolates from CF lung (our unpublished observation). Two *P. aeruginosa* strains, namely, CF1 (non-mucoid) and CF4 (mucoid), were tested in exclusion assays performed as described above. As shown in Figure 5, pre-incubation of both lactobacilli with A549 cells caused a statistically significant reduction in the CFU number of both *P. aeruginosa* strains as compared to the controls (*P. aeruginosa* incubated with A549 cells in the absence of lactobacilli), confirming the data obtained via confocal microscopy.

### 3.6. Live versus Killed LA in Preventing P. aeruginosa Adhesion to A549 Cells

**Figure 6:**
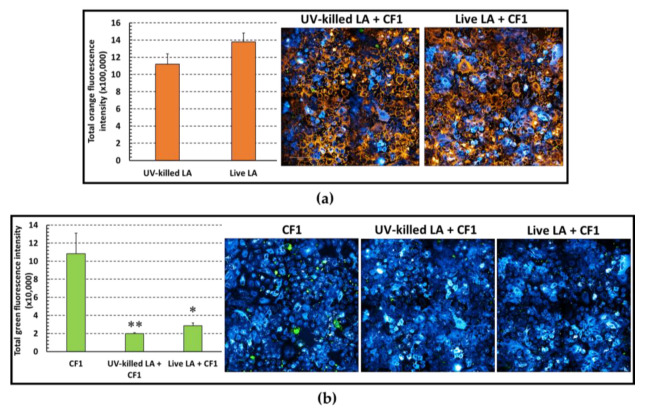
Ability of UV-killed and live LA to adhere to A549 cells and inhibit *P. aeruginosa* (CF1) adhesion analyzed via triple-fluorescence staining and confocal microscopy. A549 cells labeled in blue with the Biotracker^TM^400 were pre-incubated with live or UV-killed LA labeled in orange with PKH26. Green-labelled (PHK67) *P. aeruginosa* was then added at a MOI of 10:1. (**a**) Quantitative evaluation of orange fluorescence intensity of UV-killed and live LA incubated with A549 cells (mean values of six observations± SEM) and representative 40× confocal images. *p* > 0.05 Student’s t test. (**b**) Quantitative evaluation of green fluorescence intensity of *P. aeruginosa* adhered to A549 cells in the presence of UV-killed or live LA (n = 4, mean values± SEM) and corresponding representative confocal imaging. (*) *p* < 0.05, (**) *p* < 0.01, One way analysis of variance test followed by Tukey–Kramer multiple comparisons test.

Although probiotics are generally considered harmless microorganisms, their administration to vulnerable individuals may pose safety concerns. Therefore, we aimed to investigate whether killed LA could adhere to A549 cells and exert the same exclusion effect seen for live LA. Triple fluorescence staining of A549 cells, *P. aeruginosa* CF1, and either live or UV killed LA were performed as described above and analyzed with confocal microscopy. No statistically significant difference was observed between live and UV-killed LA in adhering to A549 cells (Figure 6a). Both UV-Killed and live LA reduced *P. aeruginosa* adhesion to the same cells in a statistically significant manner, indicating that the observed exclusion effect was not dependent on the vitality of LA (Figure 6b). 

### 3.7. Live versus Killed LA in Dampening the P. aeruginosa-Induced Pro-Inflammatory Response of Human PBMC 

**Figure 7:**
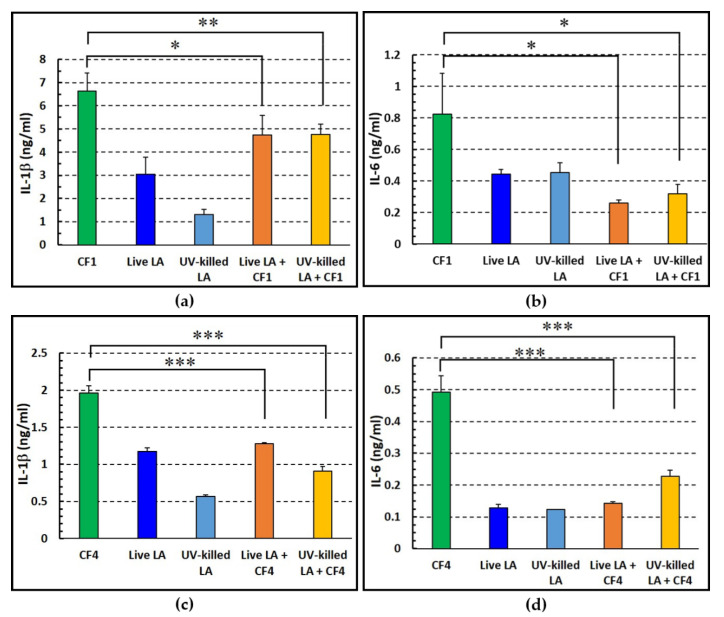
Pro-inflammatory cytokine profiles of *P. aeruginosa*-stimulated PBMCs in the presence or absence of live or UV-killed *L. acidophilus* (LA). (**a**,**c**) IL-1β; (**b**,**d**) IL-6. The figure depicts the mean values ± SEM after subtraction of the values detected for unstimulated PBMCs. CF1: non-mucoid and CF4: mucoid *P. aeruginosa* strains. (*) *p* < 0.05, (**) *p* < 0.01, (***) *p* < 0.001, One way analysis of variance test followed by Tukey–Kramer multiple comparisons test (five different donors for CF1 and a representative donor in triplicates for CF4).

PBMCs represent an important cellular infiltrate in the lung during *P. aeruginosa* infection. Therefore, we sought to evaluate the immune-modulating effect of live and UV-killed LA on human PBMCs by co-incubating them with *P. aeruginosa*, non-mucoid strain CF1, and evaluating the levels of two pro-inflammatory cytokines (IL-1β and IL-6) released in the culture supernatants. A marked pro-inflammatory effect was observed when *P. aeruginosa* alone was incubated with PBMCs, while both live and UV-killed LA elicited a mild pro-inflammatory effect (Figure 7a,b). Co-incubation of *P. aeruginosa* with LA significantly reduced the amount of both IL-1β and IL-6 released in the culture supernatants by PBMCs as compared to cells stimulated with *P. aeruginosa* alone. Live and UV-killed bacteria were similarly effective in reducing the cytokine production induced by *P. aeruginosa* (Figure 7a,b). As shown in Figure 7c,d, similar results were also obtained in a representative donor when a mucoid strain of *P. aeruginosa* (strain CF4) was used to stimulate PBMCs. 

## 4. Discussion

Despite the advent of the CFTR modulator therapies, chronic respiratory infections sustained by *P. aeruginosa* remains a major cause of morbidity and mortality in patients with CF, driving the interest in identifying innovative antimicrobial strategies to substitute or complement antibiotic use in CF [22]. The possible use of probiotics to control respiratory infections is one of these strategies, although a definitive consensus on the clinical efficacy of this type of intervention is still lacking [19]. In some studies, probiotic administration to CF patients via the oral route has been demonstrated to partially restore gut dysbiosis, reduce intestinal inflammation, and lower lung infections and exacerbation rate through mechanisms mainly attributable to the gut–lung axis. The possibility of a respiratory administration of probiotics via nasal spray or aerosol is recently emerging, suggesting that the beneficial effect of probiotics could be enhanced through their direct delivery to the infectious site [19].

Most of the studies assessing the effects of probiotics at the host–pathogen interface are conducted in the context of the intestinal environment, as this is the main final destination of beneficial microbes given via the oral route. However, probiotics reaching the lung from the intestine or via aerosol administration may experience niche-specific conditions that can greatly differ from those found in the gut. Despite this, the ability of probiotics to colonize the lung environment and compete with pulmonary pathogens is a largely un-explored area of research. Thus, in this study, we aimed to explore in vitro the interaction of a number of commonly used probiotic strains with human cells relevant for the pulmonary environment. Furthermore, we aimed to evaluate probiotic ability to compete with one of the major lung pathogens in CF, *P. aeruginosa*, and to reduce the inflammatory response induced by such pathogens in terms of cytokine release.

Ability to adhere to host cells is one of the most common selection criteria for potential probiotic strains, as it is believed to contrast mechanical removing forces (e.g., peristalsis, respiratory movements), favoring mucosal colonization and interaction with the epithelial layer [23]. This transient colonization, in turn, allows potential probiotics to stay long enough to exert their positive effects, either through direct interaction with host cells, or indirectly through the production of active metabolites. Adhesion mechanisms of probiotics to epithelial cells have been thoroughly investigated in vitro using immortalized cell lines relevant for the intestinal environment such as Caco-2 or HT-29 [24,25,26]. Such studies have suggested that, following an initial unspecific binding mainly due to hydrophobic interactions, a more specific binding phase occurs which involves the interaction of bacterial surface components with host molecules that act as receptors [23]. Bacterial components demonstrated to play a role in adhesion include lipoteichoic acid, surface-associated proteins, mucin-binding proteins, fimbriae, and pili. These latter, widely characterized in Gram-negative bacteria, have more recently also been identified in Gram-positive bacteria, including lactobacilli [27,28]. To the best of our knowledge, our study is one of the first reports investigating the adhesion ability of strains of lactobacilli to human lung epithelial cells. The results obtained reveal a differential ability of the tested strains to adhere to A549 cells, possibly reflecting differences in hydrophobicity and/or surface molecule expression across different strains. Interestingly, LA showed the highest adhesion properties, making such strains an interesting candidate for further studies aimed at evaluating the potential of probiotics in the context of pulmonary infections. LA adhesion was dose-dependent and caused cytotoxic effects only at high multiplicity of infection. The strain with the second highest adhesive capacity was LP, followed by LRm. Zawistowska-Rojek et al. recently assessed the adhesion ability of various *Lactobacillus* strains (either probiotics or clinical isolates) to Caco-2 cells [24]. Although there was a certain degree of variability across strains, even within the same species, overall, a clinical isolate of *L. plantarum* exhibited the highest adhesion ability, while the weakest adhesion was observed for *L. rhamnosus* and *L. acidophilus*, a pattern only partially matching the one observed in this study. Several parameters are reported to influence adhesion capacity of potential probiotics, including the origin of the strain, its growth phase, bacterial and host cell culture conditions, harvesting time, and intensity and number of washes of the cells to remove non-adhered bacteria [29]. All these variables render somewhat arduous the comparison among different studies. Nevertheless, we suggest that, in parallel with the widening of the field of probiotic application outside the intestinal tract, studies aimed at evaluating probiotics’ properties in site-specific conditions should be carried out, considering host cells representative of the district as a further possible variable to take into account.

According to FAO/WHO guidelines, besides having the ability to adhere to epithelial cells, potential probiotics strains must exhibit antagonistic properties against pathogenic microorganisms [30]. *P. aeruginosa* is a major respiratory pathogen [31]. In CF patients, in particular, its prevalence ranges from 10 to 30% at ages 0–5, increasing up to 80% at age ≥ 18 years [32]. The acquisition of this pathogen in CF patients is associated with a worse prognosis and a deterioration of lung function, which is the leading cause of patients’ morbidity and mortality [33]. In agreement with previous reports [34], in this study, we observed the ability of *P. aeruginosa* strains isolated from CF patients to adhere to A549 cells. Confocal microscopy experiments demonstrated that pre-treatment of A549 cells with lactobacilli reduced the association of *P. aeruginosa* to the same cells, although to different extents depending on the *Lactobacillus* strain used. In agreement with the adhesion results, LA was found to be the most efficient in preventing *P. aeruginosa* association with A549 cells, suggesting that the probiotic could occupy the surface of host cells, reducing the availability of host binding sites for the pathogen. A similar effect was observed also for LRm as assessed by CFU count. In contrast, LP, which had shown good adherence, proved scarcely effective in the exclusion assay, suggesting that ability to adhere and to prevent *P. aeruginosa* adhesion are two properties that do not necessarily coincide. The likely exclusion effect played by LA on *P. aeruginosa* was confirmed by a three-color cell adhesion assay coupled with the computer-assisted quantification of fluorescence. Such a method can simultaneously probe and localize the relative adhesion of lactobacilli and *P. aeruginosa* onto host cells, and proved to be a useful tool for studying the complex interplay between probiotics, bacterial pathogens, and host cells. In addition to saturation of binding sites, other mechanisms of exclusion played by lactobacilli may occur. For instance, it has been recently demonstrated that exopolysaccharides from *Lactobacillus delbrueckii* ssp. *bulgaricus* modulate the surface expression of a molecule used by bacteria for adherence on A549 cells (CEACAM-1), suggesting that the exclusion effect may also reflect a different pattern of host receptor expression induced by lactobacilli interaction with host cells [35]. 

Although probiotics are generally recognized as safe (GRAS), their use as prophylactic or therapeutic measure may pose a safety concern, especially in vulnerable individuals such as CF patients. Although rare, systemic infections and immune stimulation rather than suppression or metabolism alteration are side effects that have been described following probiotic supplementation via the oral route [36]. Expression of virulence factors and potential ability to transfer resistant determinants to other commensal or pathogenic bacteria present in the ecological niche are other concerns often raised against probiotic use. Thus, in the last part of our study, we wanted to assess the anti-adhesion ability of dead lactobacilli as an alternative to the use of live bacteria. To this end, LA was killed via exposure to UV light, in the attempt to preserve bacterial integrity and maintain the pattern of surface-associated molecules. Interestingly, the results obtained showed that killed, intact bacteria could prevent *P. aeruginosa* adhesion to A549 cells induced by the bacterium as efficiently as live bacteria. 

Inflammation-mediated damage of the airways significantly contributes to the pathogenesis of chronic lung infections and represents an important therapeutic target [37]. In an in vitro cell infection model, we have recently demonstrated that *P. aeruginosa* elicits a strong pro-inflammatory response which is even enhanced when the bacteria switch from the planktonic to the biofilm mode of growth [21]. Due to the recognized immunomodulatory effects played by probiotics, in this study, we sought to investigate whether LA could dampen the pro-inflammatory response elicited by a *P. aeruginosa* lung isolate towards PBMCs, as these cells represent an important cellular infiltrate in the lung of CF patients. The presence of both live and killed lactobacilli reduced the amount of pro-inflammatory cytokines released in the supernatants. Such findings might be interesting from a translational point of view, as it has been demonstrated that the pro-inflammatory response to bacterial infection is particularly exaggerated in CF patients [37]. Thus, the use of probiotics alone or in combination with other anti-inflammatory agents could help in dampening the unbalanced host response to the infection, preventing tissue damage and lung deterioration.

Overall, these results support the current view that probiotic effects go beyond their vitality [38,39] and open up new possibilities of probiotic formulations in vulnerable or immune-deficient individuals.

We believe that our study proposes a novel application approach to probiotics for the management of lung infections and opens the road to future studies and research directions. Adhesion capacity and anti-inflammatory properties of probiotics do not necessarily coincide, and it will therefore be interesting to evaluate whether strains that in the present screening exhibited low adhesion capacity may also exert detectable anti-inflammatory profiles. Likewise, identification and isolation for the probiotics structural components involved in adhesion to host cells and/or in the immunomodulatory effects is an interesting prospect with future applicative potential. Finally, evaluation of the protective effects of probiotics, parabiotics, or postbiotics in complex 3D lung infection models or in a mouse model will be a necessary step to address the potential of such preparations in vivo as an innovative strategy for the prevention/treatment of *P. aeruginosa* lung infection.

## 5. Conclusions

In conclusion, the results obtained disclosed a diversity in the adhesion properties of various strains of lactobacilli to human lung epithelial cells. Based upon such adhesive properties, LA was identified as an interesting candidate displaying the ability to inhibit the adhesion of *P. aeruginosa* to pulmonary epithelial cells (by a probable exclusion effect) and to reduce the release of pro-inflammatory cytokines from human peripheral blood mononuclear cells stimulated with *P. aeruginosa*. The use of inactivated (exposed to UV light) LA produced similar effects, consolidating the hypothesis that the structure itself of these microorganisms might be sufficient to exert beneficial outcomes similar to those obtained when they are viable. Further studies are needed to disclose the molecular mechanisms of the probiotic action of LA at the host–pathogen interface. One can hypothesize that saturation of innate immune receptors on epithelial and/or mononuclear cells by LA may prevent the pathogen’s interaction with such receptors, reducing the pathogen’s ability to adhere to host cells and stimulate an excessive pro-inflammatory response. 

The triple-fluorescence staining and the computer-assisted quantitative analysis with the Operetta system reported in this study was revealed to be a feasible method to simultaneously probe and localize the relative adhesion of probiotics and pathogens onto host cells. Such a method may represent an important tool for studying the complex interplay between bacterial pathogens, beneficial bacteria, and the host. Further studies in complex 3D lung infection models or in the mice model will provide insights on the feasibility of aerogenic administration of probiotics, either live or killed, for the prevention or treatment of *P. aeruginosa* lung-infections.

## Data Availability

The data presented in this study are available on request from the corresponding authors.
